# Does believing something to be fiction allow a form of moral licencing or a ‘fictive pass’ in understanding others’ actions?

**DOI:** 10.3389/fpsyg.2023.1159866

**Published:** 2023-05-15

**Authors:** Jacqueline Thompson, Ben Teasdale, Evert van Emde Boas, Felix Budelmann, Sophie Duncan, Laurie Maguire, Robin Dunbar

**Affiliations:** Calleva Research Centre, Magdalen College, Oxford, United Kingdom

**Keywords:** fictional transportation, fictive pass, identification, causal attribution, empathy

## Abstract

**Introduction:**

The human capacity to engage with fictional worlds raises important psychological questions about the mechanisms that make this possible. Of particular interest is whether people respond differently to fictional stories compared to factual ones in terms of how immersed they become and how they view the characters involved and their actions. It has been suggested that fiction provides us with a ‘fictive pass’ that allows us to evaluate in a more balanced, detached way the morality of a character’s behaviour.

**Methods:**

We use a randomised controlled experimental design to test this.

**Results and discussion:**

We show that, although knowing whether a substantial film clip is fact or fiction does not affect how engaged with (‘transported’ by) a troubling story an observer becomes, it does grant them a ‘fictive pass’ to empathise with a moral transgressor. However, a fictive pass does not override the capacity to judge the causes of a character’s moral transgression (at least as indexed by a causal attribution task).

## Introduction

Humans are obsessed with stories, and the art of telling stories is one of the universals of human culture, present in every part of the world and back through time for at least as far as we have written records (Gottschall, 2012). As the novelist Graham Swift says, ‘man is a storytelling animal: where ever he goes he wants to leave behind not a chaotic wake, not an empty space, but the comforting marker-buoys and trail-signs of stories’ (Swift, 2002, p. 62–63). No modern culture lacks a tradition of storytelling (oral folktales), indicating that the capacity to tell stories is clearly of very ancient origin. Indeed, some stories can have an extremely wide geographical and cultural distribution (e.g., the *Tale of the Two Sisters*: Ross et al., 2013), suggesting long cultural ancestries mediated by oral transmission.

Whilst it is easy to see that there may be value to factual stories in that they enable us to learn about new territories or how to deal with events or people we have not ourselves experienced, it is less obvious why we should find fictional stories as compelling as we evidently do. After all, the action in a fictional story is not happening to *real* people. Amongst the functions that have been suggested for fiction have been learning social skills without risk (Mar et al., 2006; Kidd and Castano, 2013), providing a context for experiencing rewarding states or processing upsetting situations (Bloom, 2008) and community bonding (leading to enhanced cooperation) through the recitation of origin stories and folk myths (Dunbar, 2005; Smith et al., 2017), none of which are, of course, necessarily mutually exclusive. Irrespective of the benefits of story-telling, the fact that we are prepared to invest so much time, effort and money in reading, listening to or watching fictional books, plays and films – and, in some cases, the same story over and over again—raises deep psychological questions (Tan, 2018).

Our capacity to become immersed in (or transported by) stories, and especially fictional stories, is self-evident. Although individuals may vary in the extent to which they experience this, we typically lose track of time, are not aware of the environment around us, and can become so completely wrapped up in a character’s actions or predicament that we experience the same emotional responses as we would if it was actually happening to us. In the wider literature, this state is referred to variously as ‘transportation,’ ‘immersion,’ ‘absorption,’ ‘narrative engagement,’ or ‘the literary illusion’ (Gerrig, 1993; Green and Brock, 2000; Green et al., 2004; Green, 2005; Busselle and Bilandzic, 2009; Wolf et al., 2013; Kuijpers et al., 2014; van Laer et al., 2014; Ryan, 2015); here, we will use the terms ‘immersion’ and ‘transportation’ interchangeably. The more important issue is that, psychologically, the capacity to become transported in this way is remarkable because what we experience is not triggered by the physical world acting on us. In fact, to be able to do this, we need to be able to inhibit the real world in which we are unavoidably immersed so as to be able to imagine the experiences of protagonists in a story world that does not actually exist. This is no trivial cognitive task (Viard et al., 2011; Lewis et al., 2017).

Two important questions about the psychology of fiction are, thus, whether and how our responses to fiction differ from those to factual stories. Goldstein (2009), for example, found that fiction arouses the same emotional response (in this case, sadness) as factual material, without the anxiety that real life events would normally elicit. Interestingly, Sperduti et al. (2016) found this difference only in respect of verbal response ratings, and not in galvanic skin responses (electrodermal activity reflecting underlying physiological responses). A number of studies have reported that written texts, short film clips and even photographs labelled as ‘real’ evoke more emotion, whereas those labelled as ‘fiction’ evoke more thought and reflection (Argo et al., 2007; Mendelson and Papacharissi, 2010; Igartua and Vega Casanova, 2016; Sperduti et al., 2016).

How effectively we engage in this way may depend on the level of immersion or transportation that individuals experience when watching or listening to a story. Individuals might, for example, become less immersed in a factual story if they are appalled by the events described, but be able to engage more when they are aware that it is ‘simply a story’ (Gottschall, 2012). This might result in morality judgements differing between reality and fiction. Sabo and Giner-Sorolla (2017), for example, found that characters in a short vignette were more likely to be given a ‘fictive pass’ for harmful acts (anger) in ‘fictional’ compared to ‘real’ situations, although this was not true for ‘impure acts’ such as those involving disgust. This might enable the audience to take a more detached view of the characters than they would of real life events and so take a more balanced moral stance. In effect, they may be able to evaluate the moral rights and wrongs of different behaviours without being burdened by the confounding effects of moral partiality (the tendency to excuse the moral failings of close family and friends: Cottingham, 1986), thereby correctly identifying the moral blame (*sensu* causal attribution: Kinderman and Bentall, 1996) that should be attached to the action. This might also give rise to something analogous to ‘moral licensing’ (Merritt et al., 2010; Blanken et al., 2015) in that, in a fictional context, we may be allowed to empathise with someone who has broken a moral code in a way that would not be allowed in real life.

Of course, these responses are likely depend in part on an individual’s psychological traits. Engaging with fiction is cognitively demanding because it requires us to model events and, especially, characters’ mental states in the virtual statespace of our mind. Mentalising (imagining how someone else might feel when it is not actually happening to us) is neurophysiologically demanding: it requires the differential recruitment of neurons in the brain’s default mode neural network (Lewis et al., 2017). Individuals differ considerably in their mentalising abilities, and enjoyment of, and immersion in, stories of different mentalising complexity is known to be influenced by individuals’ mentalising competences (Carney et al., 2014). The degree of transportation might also depend on aspects of personality. Rain and Mar (2021), for example, found that ‘parasocial interaction’ (engagement with a character in, say, a film: Horton and Wohl, 1956) is influenced differentially by an individual’s attachment style, in particular the anxiety dimension in high avoidant individuals (see also Rain et al., 2017).

In this study, we ask whether observers respond differently if a film clip is framed either as reality or as fiction. Many previous studies have used short (5–10 s) clips as their experimental stimuli. However, short clips may not allow full immersion, so we here use an extended 25-min segment of video film of a real-life event (a police interview of a murder suspect, culminating in admission of guilt). Crucially, this allows us to compare audience response to an unfolding narrative drama, thereby providing greater ecological validity. Visch et al. (2010) showed that emotional responses to brief film clips were independent of the film itself, whereas how participants categorised the film depended on the cognitive interpretation of context, and hence on a level of cognitive detachment. By analogy, we suggest that viewers’ automatic (generally, emotional) responses to the video will be similar regardless of their belief as to whether the video is real or fiction, but that their cognitive responses will differ as a function of their belief.

Our interest lies in whether or not the framing given to a drama (fiction vs. real) allows the observer to become more immersed in the story, and whether this affects the viewer’s moral attitude towards, and willingness to identify (i.e., empathise) with, someone who has transgressed a moral code. In effect, does fiction grant the observer a ‘fictive pass’ in a way that does not happen when we know that the story is true? A follow-up question is whether immersion in the story causes us to adopt a more sympathetic attitude and so derails our ability to identify the cause in a detached way for a moral transgressor’s action, to understand why the suspect behaved as he did (causal attribution: Kinderman and Bentall, 1996).

Specifically, we test the following hypotheses:

(H1) Attention, transportation, enjoyment and emotional engagement should not differ between framing conditions (Real vs. Fiction) if these primary (essentially physiological) responses are triggered by how emotionally engaging the film is and not by whether it is fact or fiction. If participants are disproportionately engaged by one condition compared to the other, that would make it difficult to interpret the significance of any differences in the variables of interest (hypotheses H2 and H3).

(H2) Notwithstanding one’s moral attitudes towards murderers in general, either in real life or in fiction (one’s ‘moral compass’), emotional identification with the protagonists in the story will be higher in the fiction condition than in the ‘real life’ condition because observers should be less appalled by a fictional murder than an actual murder (i.e., can exploit a *fictive pass*). Conversely, the extent to which participants are appalled by Williams’ actions should be lower in the ‘Fiction’ condition if this allows a ‘fictive pass.’

(H3) Because of the background information provided in the video (e.g., that Williams turns out to be a serial killer), we predict that, in both conditions, participants will be more likely to attribute the murders to Williams’ personality (internal/personal); in contrast, because of his evident concerns for his wife and the reputation of his employers (the Royal Canadian Air Force), they should be more likely to attribute his behaviour in the interview (i.e., his confession to the murders) to social context (external/personal) rather than the situation he finds himself in (external/situational). If fiction allows a ‘fictive pass,’ then we might expect participants in the ‘Fiction’ condition to exhibit a wider range of explanations compared to those in the ‘Real’ condition as a result of being able to take greater moral sympathy with him, whereas natural horror responses to an actual murder (real condition) might be more likely to cause the murderer to be held entirely to blame for his actions.

## Methods

### Participants

A total of 123 participants (76 female; mean age 29.2 ± 11.30SD, range 18–60 years; 78% white, 11.4% Asian, 3.3% black, 6.5% mixed ethnicity) were recruited via university mail lists and posters. Participants were required to be native English speakers. Participants were paid £10 for taking part.

Participants were randomly assigned to one of two conditions (‘Real’ or ‘Fiction’) in the order they signed on to book an experimental slot. On arrival, participants in the ‘Real’ condition were informed that they were about to watch a video recording of a real-life police interview of a murder suspect (see following section); those in the ‘Fiction’ condition were informed that they would be watching a fictional enactment of an interview by professional actors. However, it became apparent during post-experiment manipulation checks that a number of those assigned to the ‘Fiction’ condition suspected that the interview was actually real. These individuals were removed from the analysis and treated separately. Since this meant a significant shortfall in the ‘Fiction’ condition sample, additional participants were subsequently recruited to this condition. With these additional samples, there were 44 and 42 participants for analysis in the ‘Real’ and ‘Fiction [believer]’ conditions, respectively, with 37 in the ‘Fiction [unconvinced]’ group (Table 1). The two conditions used in the analyses did not differ in age (t_85_ = 0.86, *p* = 0.395), gender (χ^2^ = 1.19, df = 1, *p* = 0.550) or ethnicity [white vs. non-white: (χ^2^ = 1.59, df = 1, *p* = 0.208)].

**Table 1:** Statistical results for manipulation checks.

Question	Real	Fiction/unconvinced	Fiction/believer	F^*^	df	*p*
	Mean ± SD	Mean ± SD	Mean ± SD			
Were you aware of the Williams case? [0–5 = no]	4.73 ± 0.76	4.97 ± 0.17	4.79 ± 0.56	0.17	1.84	0.679
Believed video was fictional [0–100 = fiction]	14.4 ± 15.4	15.0 ± 16.5	49.60 ± 33.1	40.81	1.82	<0.001
How realistic did the video seem? [0–100 = real]	88.02 ± 14.4	84.73 ± 20.6	78.42 ± 23.3	5.29	1.83	0.024
How upsetting was the video? [0–100 = very]	43.14 ± 26.0	43.40 ± 23.1	49.52 ± 26.5	0.00	1.84	0.962
Did you suspect it was real [1–5 = real]	3.80 ± 0.41	3.75 ± 1.36	1.45 + 0.50	578.1	1.85	<0.001
Sample size	44	37	42			

* Comparison between real vs. fiction [believed] conditions only; all *p*-values 2-tailed.

The experiment was time-consuming to run (it took well over an hour in each case, and only one participant could be run at a time), a sample of 80 divided between the two conditions was considered adequate in terms of power to detect a relatively strong effect in the light of similar previous experiments (see Teasdale et al., 2021).

### Stimulus

We use a 25-min clip edited from the publicly available video of an actual police interview that had been conducted on 07 February 2010 during the investigation of a Canadian multiple murder. The clip involves only the suspect (Russell Williams, a senior officer in the Royal Canadian Air Force) and a detective (Detective Staff Sergeant Jim Smyth) who interviews him. In this context, the detective provides us with a morally neutral baseline against which to judge responses to Williams as a murderer. During the course of the video (filmed from four fixed camera angles, all visible in split-screen format), the detective gradually presents, in a very calm way, more and more circumstantial and factual evidence to Williams until eventually he confesses. In total, the full video lasts ~3 h and is the last of a number of separate interviews. The clip only shows the interior of a bare interview room; there are no pictures of the victim, the murder scene or any of the evidence (other than that obliquely visible when Smyth shows Williams photographs of bootprints and tyre tracks at the crime scene). Even though unscripted, and heavily condensed, the edited video has a compelling narrative arc, as Russell slowly but surely loses ground and is finally led to confess. A summary of the action is given in the online Supplementary material. The full 25-min video clip as shown to participants in the experiment is available at: https://youtu.be/f0254BvJVXc. The entire full length interview can be accessed at: https://www.youtube.com/watch?v=hzh3adTWZOg,watch?v=Ah51vPzcVEM,watch?v=2mQA2yQFZ8o.

The crucial element in the experimental design was the framing given to participants at the outset indicating that the video they were about to watch was either factual or fictional. Real life conversations/interviews typically have a slow pace with long pauses; in order to maintain dramatic pacing and to avoid wasting screen time that could be better used for other parts of the action, fictionalised scripts typically compress the time by omitting most of the natural silences and speeding up the flow of the dialogue. Since this might provide cues as to whether a video clip was real or fictionalised, we provided a cover story in the fiction condition suggesting that the clip was taken ‘from an original web series which creates a realistic feel by using techniques such as fixed-camera (CCTV-style) shooting, as well as asking the actors to improvise their scenes from a minimal script.’ The introductory text for the fiction condition also explicitly referred to the video as ‘fictional’ and gave a credits list for the ‘actors’ as well as a ‘director’ and ‘production company.’ The text for the two frames can be found in the online Supplementary material.

### Procedure

After completing consent forms and providing demographic data, participants watched the video clip alone on a computer in a quiet room and then answered a series of questionnaires designed to probe their enjoyment of the film and their response to the manipulation, as well as other background information. We used the following standardised instruments:

To test hypothesis H1:

- Story World Absorption Scale, or SWAS (Kuijpers et al., 2014), to measure absorption into (transportation by or immersion in) the film. The SWAS comprises 4 subscales, 3 of which we deemed relevant to the medium of film: attention (e.g., ‘When I finished the film I was surprised to see that time had gone by so fast’), transportation (e.g., ‘When I was watching the film it sometimes seemed as if I were in the world of the film too’), and emotional engagement (e.g., ‘I felt how the main character was feeling,’ ‘I felt sympathy for the main character’), each rated on a 1–7 (7 = strongly agree) Likert scale.
- A 4-question enjoyment scale (Likert scale 1–7, 7 = strongly agree; adapted from Dunbar et al., 2016) to determine how much they enjoyed the film: the scale is a set of simple factual statement about enjoyment, not a rating of beliefs. The questions are given in the *SI*.

To test hypothesis H2:

- A 6-question ‘moral compass’ scale to probe how moral the participant thought it was to identify or sympathise with a murderer (Likert scale 1–7, 7 = strongly agree). The questions are given in the *SI*.
- A 7-question identification scale (Likert scale 1–7, 7 = strongly agree; from Cohen, 2001 and Tal-Or and Cohen, 2010) with respect to each protagonist to measure the extent to which participants identified with the characters in the story (i.e., that we forget ourselves and become the character in the story, thereby feeling that the events in the story are happening to us: Cohen, 2001). We used only the first five items from this questionnaire because the last two questions are concerned with moral views rather than immersion. The scale as used in the experiment is given in the *SI*.
- Two simple factual questions asking whether the participant morally approved of Russell’s behaviour (a) during the interview and (b) in the light of the crime he had committed (Likert scale 1–7, 7 = strongly agree). The questions are given in the *SI*.
- Two simple questions asking how disgusted and angry the participant felt about Williams after he had confessed (Likert scale 1–7, 7 = very disgusted/angry). The questions are given in the *SI*.

To test hypothesis H3:

• The IPSAQ attributional scale of Kinderman and Bentall (1996) to assess participants’ perception of where the cause or blame for (a) the confession and (b) the murders lay. Attribution is conventionally differentiated between three subscales: Internal/Personal (in the context of the interview, Williams’s realisation that he was caught and had better own up; for the murders, his psychological predisposition to stalk young women, his sexual desires), External/Personal (e.g., Williams’s wish to protect his wife, Detective Smyth’s skill at interrogation and the police force’s work at matching up tyre tracks and footprints) and External/Situational (i.e., circumstances or chance, such as the nerve-wracking environment of being in an interrogation room; the police’s luck at finding the tyre tracks before the snow melted). We adapted this instrument to a single question for each of the three subscales: ‘Was Williams’ behaviour due to [his own personality/his personal social circumstances/the situation he found himself in]?,’ scored on a 1–7 (7 = strongly agree) Likert scale in each case. These are intended to ask simply whether participants place the locus of blame on Williams’s personality, the social circumstances within which Williams is immersed or the situational context over which Williams had no control.

In addition, participants were asked how often they thought crimes of this kind happened in Canada (‘How often to you think crimes like Williams’s occur in Canada [in real life]?’) with replies on a 6-point Likert scale (1 = never; 6 = all the time), and whether or not they had heard of the Williams murder case (on a 5-point Likert scale, 1 = definitely yes and 5 = definitely not). Only 1 participant indicated that they had heard about it, but, as it happened, this participant had been allocated to the ‘Real’ condition anyway. Four other participants answered ‘not sure,’ and all others responded ‘probably not’ or ‘definitely not.’ Since the number who had heard of the case was very small (<1%), and the only one that was fully aware of it was assigned to the Reality condition, there was no reason to exclude any individuals from the analyses. Overall, there was no statistical difference in the ratings for prior familiarity with the murder case between the three conditions (Table 1: *F*_2,119_ = 2.04, *p* = 0.134).

### Manipulation check

As a manipulation check, we asked participants four increasingly specific questions as part of a funnel debriefing (see Table 1). On a sliding scale (0 to 100), these were:

1. How realistic did the video seem? [100 = completely fictional]
2. When you were watching the video, how much did you believe the video was fictional (acted drama) vs. real (real-life footage)? [100 = completely fictional]
3. How upsetting did you find the video? [100 = very upsetting]

The fourth question gave more context (tailored to the instruction condition) and collected responses in a multiple-choice 5-point scale (1 = complete belief; 5 = complete disbelief):

4. Before watching the video, you were told that it was [from an acted tv drama/real interrogation footage]. Whilst watching the video and answering the questions, to what extent did you suspect that the video was not actually [fictional drama/real footage] as you had been told? [scale for both conditions transposed to 100 = believed it was completely fictional]

We used the fourth question as our main manipulation check, as it was the most detailed and least open to ambiguous interpretation. However, the third question was added only after the first 10 participants had already taken part, when participant anecdotal feedback made it clear that the other manipulation check questions could be misinterpreted. Nevertheless, a simple linear regression analysis showed that the sliding scale response for question 2 (‘how much did you believe the video was fictional (acted drama) vs. real (real-life footage)?’) was in fact a strong predictor of the ordinal multiple-choice responses in the other 113 participants. Therefore, for the first 10 participants, we calculated the mean sliding scale ratings for each multiple-choice response, and assigned the multiple-choice response with the mean sliding scale rating numerically closest to their own sliding scale response (we did this separately for real and fiction conditions). To be safe, we ran two versions of all analyses including and excluding the first 10 participants as a factor. The results did not differ in any way.

Table 1 summarises the results for the manipulation checks, partitioning the ‘Fiction’ condition into those who stated afterwards that they believed the video had been fictional drama and those who declared that they suspected it was actually real. Those in the ‘Real’ condition were significantly less likely to think the video was fictional than those in the ‘Fiction’ group, and were more likely both to think it was a real police interview and to consider it realistic. Whilst a majority of participants in the ‘Fiction’ condition indicated that they were convinced by the deception, 44% of those initially assigned to this condition indicated that they had at least some doubt, and these participants (labelled ‘Fiction/unconvinced’ in the tables) were excluded from the formal analyses, although we provide their data. There was, however, no difference between the two conditions in how upsetting they found the video to be. In sum, those in the ‘Fiction(believed)’ condition did believe the cover story and supposed they were watching a fictional account. Whilst those in the ‘Real’ condition were on average somewhat less emotionally aroused by the video, nonetheless the difference between the two conditions was not significant. Thus, the video elicited the required responses.

### Data

The data are provided in the online Supplementary material file *Real-Unreal_ Dataset.csv*.

## Results

The manipulation checks established both that participants did believe they were watching either a true life recording or a fictional improvisation, depending on the framing they had been given (i.e., the manipulation worked), and that they were equally emotionally aroused by the video (hence any differences in other responses will not reflect different levels of arousal). Hypothesis H1 then sought to establish whether fictional stories engross/transport us more than factual ones, with an underlying expectation that, if these largely emotional/physiological responses were driven by the content and pacing of the film and not by the framing condition, there would be no differences in response between the two conditions. Hypothesis H2 tested the prediction that, even in the absence of any difference in immersion/transportation, measures of identification and moral engagement with the two characters (but especially the murderer, Russell Williams) would nonetheless be very different in the two conditions because the ‘fiction’ label provides a fictive pass allowing viewers to engage with the murderer without feeling guilty. Finally, hypothesis H3 tested the prediction that participants in the ‘Fiction’ condition would take a more balanced view of what drove Williams to act as he did and would hence come to a wider range of opinions, whereas those in the ‘Real’ condition would be dominated by their natural gut response to a murder and would be naturally more likely to blame his own internal psychological make-up and not his circumstances.

*H1*: Engagement, transportation and enjoyment

**Figure 1:**
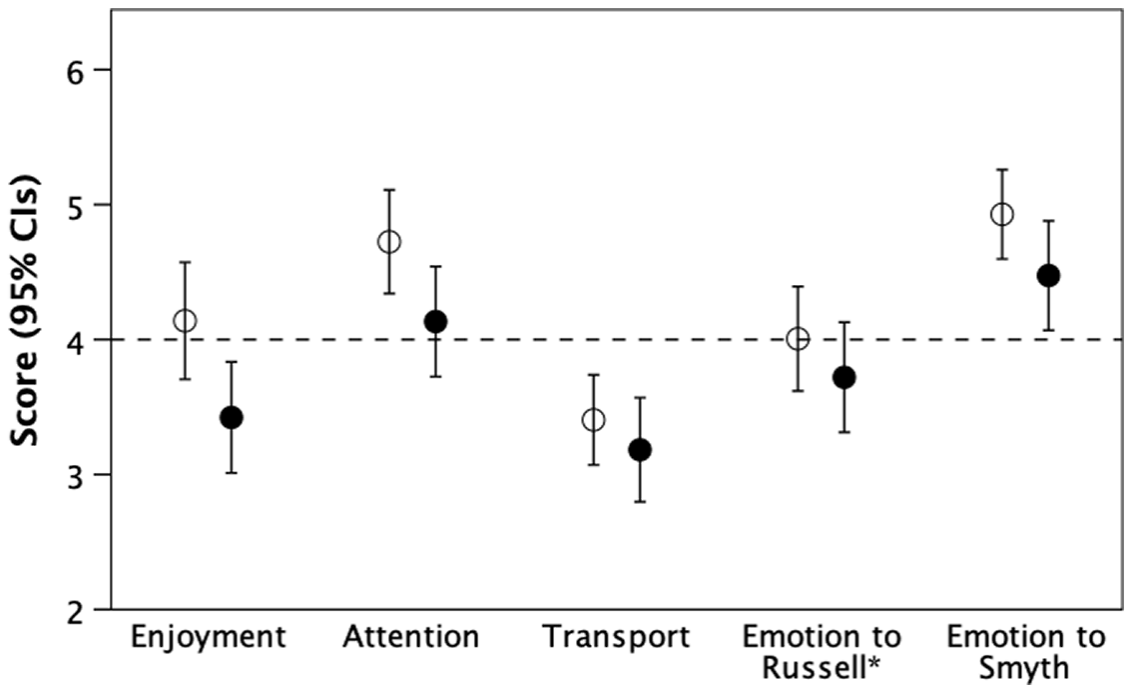
Mean (±95% CIs) for the tasks used to test hypothesis H1: transportation (immersion) whilst watching the video. Filled symbols: ‘Fiction’ condition; unfilled symbols: ‘Real’ condition. Dashed line demarcates the midpoint in the scale used. * indicates murderer.

Figure 1 plots the results, and Table 2 summarises the analyses, for the key variables that test hypothesis H1.

**Table 2:** Testing hypothesis H1: measures of immersion and transportation for two conditions.

Trait	Real		Fiction/unconvinced		Fiction/believer		t^†^	df	*p*	Cohen’s d
	Mean^*^	SD	Mean^*^	SD	Mean^*^	SD				
Enjoyment	4.14	1.44	3.67	1.66	3.42	1.32	2.42	85.0	0.018	0.517
SWAS: attention	4.72	1.28	4.53	1.15	4.13	1.31	2.13	84.2	0.036	0.457
SWAS: transportation	3.40	1.11	3.55	1.16	3.18	1.24	0.87	82.4	0.385	0.188
SWAS: emotional engage/Williams	4.01	1.29	3.82	1.53	3.72	1.31	0.58	68.4	0.562	0.220
SWAS: emotional engage/detective	4.93	1.10	4.51	1.39	4.47	1.31	1.49	65.9	0.142	0.377

^*^Rated on scale 1–7 (1 = most negative; 7 = most positive); ^†^real vs. fiction/believer, with unequal variances; all *p*-values 2-tailed.

Compared to those in the ‘Real’ condition, participants in the ‘Fiction’ condition enjoyed the video less (*t*-tests, with unequal variances: *t*_85.0_ = 2.42, *p* = 0.018) and said they paid less attention (*t*_84.2_ = 2.13, *p* = 0.036). In contrast, there were no group differences for transportation (*t*_82.4_ = 0.87, *p* = 0.385) or emotional engagement (either with Williams or with the detective; *t*_68.4_ = 0.58, *p* = 0.552 and *t*_65.9_ = 1.49, *p* = 0.142, respectively), although both clearly preferred the detective (Smyth) to the murderer (Russell).

Taken together, these results provide some support for hypothesis H1: at least for this particular type of story (crime genre), fictional stories do not necessarily lack ‘drawing’ power compared to true stories (or, to put it another way, do not necessarily need the skills of the film director to make the storyline engaging). Both can engross us. Nonetheless, it is clear that those in the ‘Fiction’ condition did not find the video quite as engaging as those in the ‘Real’ condition. Most likely this is because a real life interview lacks the speed and pace to come across as a convincingly engaging fictionalisation (indeed, at least one participant in the Fiction/unconvinced group made exactly this point). The important finding in the present context, however, is that the experimental manipulation did not disproportionately favour the Fiction condition; if anything, it favoured the ‘real life’ condition.

*H2*: Identification and moral acceptability

**Figure 2:**
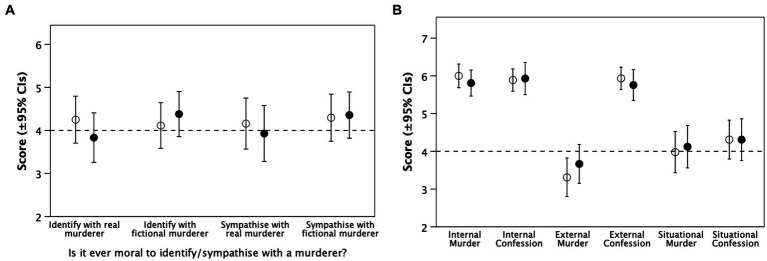
Mean (±95% CIs) for the tasks used to test hypothesis H2. **(A)** Moral compass attitudes: is it morally acceptable to identify with or feel sorry for murderers, either in real life or in fiction? **(B)** Emotional attitude towards either the detective (Smyth) or the murderer (Williams). Filled symbols: ‘Fiction’ condition; unfilled symbols: ‘Real’ condition. Dashed line demarcates the midpoint in the scale used. * indicates murderer.

Figure 2 plots the results, and Table 3 summarises the analyses, for the variables that test hypothesis H2.

**Table 3:** Testing hypothesis H2: measures of moral approval and identification for the two conditions.

Trait	Real	Fiction/unconvinced	Fiction/believer	t^†^	df	*p*	Cohen’s d
	Mean ± SD^*^	Mean ± SD^*^	Mean ± SD^*^				
Moral to identify with a real-life murderer	4.25 ± 1.79	4.06 ± 1.89	3.83 ± 1.85	1.06	84.0	0.292	0.229
Moral to identify with a fictional murderer	4.11 ± 1.74	4.25 ± 1.70	4.38 ± 1.68	−0.72	84.0	0.471	−0.156
Moral to feel sorry for a real-life murderer	4.16 ± 1.95	3.97 ± 1.68	4.30 ± 2.09	0.53	84.0	0.599	0.114
Moral to feel sorry for a fictional murderer	4.30 ± 1.80	4.42 ± 1.56	4.36 ± 1.72	−0.16	84.0	0.871	−0.035
Identified with Smyth^§^	5.10 ± 1.04	4.70 ± 0.95	4.76 ± 0.73	1.42	79.9	0.158	0.383
Identified with Williams^§^	2.87 ± 1.11	3.11 ± 1.06	3.36 ± 1.18	2.10	83.7	0.039	−0.432
Moral approval of Williams^§^	3.53 ± 1.47	4.06 ± 1.06	3.60 ± 1.40	−0.20	85.0	0.841	−0.043
Disgust at Williams^§^	5.42 ± 1.83	5.11 ± 1.47	5.55 ± 1.78	−0.32	84.8	0.747	−0.069
Anger at Williams^§^	4.38 ± 2.07	4.71 ± 1.90	4.81 ± 1.63	−1.09	82.7	0.281	−0.231

^*^Rated on scale 1–7 (1 = negative; 7 = positive); ^‡^Comparison of real vs. fiction/believer with unequal variances; all *p*-values 2-tailed. § Smyth, detective; Williams, murder suspect.

We consider first the abstract moral acceptability of identifying with a real-life vs. a fictional murderer (Figure 2A). Although in neither conditions did participants differ significantly from a random response (dashed line: *p* ≥ 0.292), they did tend to differ in their attitudes towards a real and a fictional murderer. Viewers who believed the video was fictional rated the acceptability of identifying with a fictional murderer as significantly higher than the acceptability of identifying with a real-life murderer (*t*-test: *t*_41_ = 3.27, *p* = 0.002), but there was no such difference for viewers who believed the video was real (*t*_43_ = 0.69, *p* = 0.498). In contrast, there were no significant differences in respect of the morality of feeling sorry for (sympathising with) a fictional vs. a real-life murderer (*p* ≥ 0.599).

Figure 2B considers participants’ emotional (i.e., empathic) engagement with Smyth and Williams. The two conditions did not differ in their emotional engagement (‘identify’) with either Smyth (the detective) or Williams (the murderer; *p* ≥ 0.084), but those in the ‘Fiction’ condition did identify significantly more strongly with Williams than the those in the ‘Real’ condition (*p* = 0.039); in contrast, the reverse was the case in their identification with Smyth, although the difference was not significant (*p* = 0.158). There were no differences in their moral approval of Williams’ crime (which was low) or their disgust or anger at Williams’ actions (which were both high; *p* ≥ 0.281).

**Figure 3:**
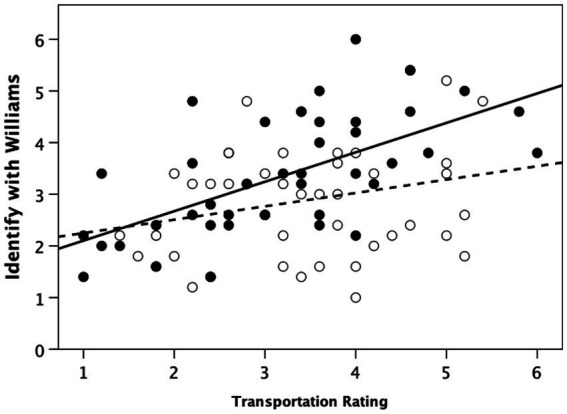
Individual emotional identification with Williams plotted against self-rated degree of transportation by the video. Filled symbols and solid regression line: ‘Fiction’ condition; unfilled symbols and dashed line: ‘Real’ condition. The regression equation for the ‘Fiction’ condition is significant, but not that for the ‘Real’ condition.

Because previous studies have indicated that participants vary in the degree to which they become immersed in (or transported by) a story, and that this can influence other correlated results (Dunbar et al., 2016), we plotted individuals’ emotional engagement with Williams (indexed as the mean of the five questions for the Identification scale with respect to Williams) against their self-rated score for transportation whilst watching the film. Figure 3 plots the results. Although there is a positive correlation for participants in the ‘Real’ condition, the regression is not significant (r^2^ = 0.068; *F*_1,43_ = 3.13, *p* = 0.084). However, that for the ‘Fiction’ condition is highly significant (r^2^ = 0.388; *F*_1,39_ = 24.75, *p* < 0.0001). Note that the two regression equations do not differ in their intercept (*t*_82_ = 1.00, *p* = 0.322), but do differ significantly in slope (*t*_82_ = 4.39, *p* < 0.0001). In other words, both groups behaved similarly unenthusiastically when they were not especially transported by the story, but the ‘Fiction’ group became much more engaged with Williams as they became more immersed in the story. This strongly suggests that a fictive pass is being enabled.

We next examined the correlation (by condition) between participants’ actual identification with the murderer and their judgement of the moral acceptability of doing so (Supplementary Figure S1). In the ‘Real’ condition, only the relationship with a real murderer was significant (Supplementary Figure S1A: r = 0.410, *N* = 44, *p* = 0.006; Supplementary Figure S1B: r = 0.147, *N* = 44, *p* = 0.341), whereas in the ‘Fiction’ condition identification with Williams was significantly correlated with the moral acceptability of identifying with both fictional (Supplementary Figure S1C: Pearson correlation, r = 0.350, *N* = 41, *p* = 0.025) and real-life (Supplementary Figure S1D: r = 0.355, *N* = 41, *p* = 0.023) murderers. The correlations between identification rating and whether it is morally acceptable to feel sorry for a murderer exhibited a clearer division between the ‘Fiction’ and ‘Real’ conditions (Supplementary Figures S2A–D: ‘Real’ condition: (a) fictional murderer, r = 0.078, *p* = 0.613; (b) real-life murderer, r = 0.145, *p* = 0.349; ‘Fiction’ condition: (c) fictional murderer, r = 0.402, *p* = 0.009; (d) real-life murderer, r = 0.436, *p* = 0.004). Once again, the ‘Fiction’ condition seems to allow a fictive pass.

Taken together, these results indicate that, as suggested by H2, moral licencing does play a role in the willingness to identify with, and hence feel sorry for, a fictional character, as well as in the willingness to morally approve of their actions. Though there are some contrary results (notably the strong correlation between identification with and moral approval of a real-life murderer in the ‘Real’ condition), broadly speaking these results confirm hypothesis H2: fiction does seem to allow a ‘fictive pass.’

*H3*: Attributing blame

**Figure 4:**
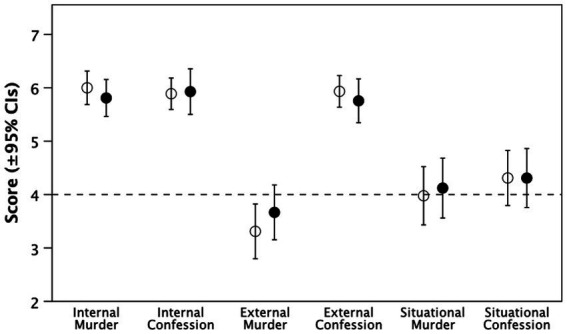
Mean (±95% CIs) for the tasks used to test hypothesis H3: understanding causal attribution for Williams’ behaviour indexed by the IPSAQ attitudinal scale, with respect to both the actual murder itself and Williams’s confession to the murder during the interview. Filled symbols: ‘Fiction’ condition; unfilled symbols: ‘Real’ condition. Dashed line demarcates the midpoint in the scale used. Internal = internal/personal factors (personality); External = external/personal (social context); Situational = external/situational (circumstances).

Figure 4 plots the results, and Table 4 summarises the analyses, for the three subscales of the IPSAQ attribution questionnaire as tests of hypothesis H3.

**Table 4:** Testing hypothesis H3: IPSAQ attribution subscales for the two conditions.

Attribution subscale		Real	Fiction/unconvinced	Fiction/believer	t^†^	df	*p*	Cohen’s d
		Mean ± SD^*^	Mean ± SD^*^	Mean ± SD^*^				
Internal/personal:	For the murder	6.00 ± 1.04	5.92 ± 0.97	5.81 ± 1.11	0.82	83.6	0.413	0.177
	Interview/confession	5.89 ± 0.98	5.09 ± 1.60	5.93 ± 1.37	−0.16	74.0	0.878-	0.034
External/personal:	For the murder	3.31 ± 1.70	3.28 ± 1.50	3.67 ± 1.65.	−0.99	84.9	0.325	0.212
	Interview/confession	5.93 ± 0.99	5.78 ± 1.02	5.76 ± 1.30	0.71	74.4	0.482	0.155
External/situational:	For the murder	3.98 ± 1.82	4.11 ± 1.74	4.12 ± 1.78	−0.37	83.6	0.711	−0.080
	Interview/confession	4.31 ± 1.77	4.29 ± 1.74	4.31 ± 1.72	0.00	84.1	0.997	0.001

^*^Rated on scale 1–7 (1 = negative; 7 = positive); ^†^Comparison of real vs. fiction/believer with unequal variances; all *p*-values 2-tailed.

As predicted, there was a strong tendency for both conditions to view Williams’s own character and psychological predispositions for both the murder and the way he handled himself in the interview. In addition, participants in both conditions considered external personal factors to be an explanation for his performance in the interview but not in respect of the murder. In contrast, neither condition viewed situational factors as having a significant role: none of the differences between the two conditions was significant.

To check whether this was simply a case of a fundamental attribution error (in this context, invariably blaming the actor’s character rather the context), we pooled the two conditions and compared ratings for personal vs. situational ratings with respect to the murder itself and to Williams’s behaviour in the interview. Participants were significantly more likely to view responsibility for the murder as being the result of Williams’s own character failings rather than due to externalities beyond his control (paired samples *t*-tests: internal vs. external/personal, *t*_85_ = 11.62, *p* < 0.0001; vs. external/situational, *t*_85_ = 8.52, p < 0.0001). However, they took a decidedly more balanced view in respect of his behaviour in the interview (internal vs. external/personal, *t*_86_ = 0.28 *p* = 0.782; vs. external/situational, *t*_87_ = 6.99, *p* < 0.0001).

On balance, this suggests that the fictive pass did not result in any kind of moral partiality effect. That participants were close to unanimity in their view that the murderer himself (whether real or fictional) was to blame for the murder would seem to be beyond a simple attribution error explanation: after all, he did admit to doing the murder and nothing in his circumstances (insofar as these emerged during the video) forced him to do it. This suggestion is reinforced by the contrast in how participants rated his performance in the interview: they took a more balanced view between Williams’s character and his personal circumstances in the context of the interview. This suggests that they were viewing each option in a reasonably neutral way rather than blaming him for everything simply because of the awful crime he had committed. To this extent, hypothesis H3 is partially supported, albeit with no evidence for the influence of a ‘fictive pass.’

### Checks for confounds

To check that our results were not simply due to participants in the ‘Real’ condition having their interest piqued by seeing something they considered rare or unusual, we examined correlations (by condition) between enjoyment or attention and participants’ ratings of the rarity of real-life murders, confessions, and publicly available police interrogation videos. We reasoned that if perceived rarity was driving the results, it ought to be correlated with enjoyment and attention in the real condition (although not necessarily in the fiction condition), such that perception of the video and its events as being rare or unusual would predict higher enjoyment and attention (i.e., morbid fascination). We found no evidence to support this hypothesis. The only correlation that even approached significance was that between enjoyment and the estimated frequency of real-life murders in Canada for participants in the ‘Real’ condition (Pearson correlation: r_45_ = 0.28, *p* = 0.06; all other *p* > 0.24), but this was in the opposite of the predicted direction (i.e., lower perceived frequency of murders was associated with *lower* enjoyment). Correlations across the pooled sample (*N* = 87) were similarly not significant (all *p* > 0.75).

We also checked whether the manipulation had differential group effects on the rarity measures: perceptions of how often murders occur in Canada, how often murderers confess to the police in interrogations, or how many real-life (and how many fictional) police interrogations are made available to the public. Ratings of the availability of fictional police interrogations were equally high across groups (*t*-tests, unequal variances: *t*_79.79_ = 0.14, *p* = 0.884), but participants in the ‘Real’ condition rated the public availability of real-life police interrogation videos as significantly higher (means: M = 3.5 on a 6-point scale) compared to participants in the ‘Fiction’ condition (M = 3.0; unequal *t*-tests: *t*_81.27_ = 2.15, *p* = 0.035). Both groups gave similar estimates of how frequently murders occur in Canada (*t*_83.78_ = 0.74, *p* = 0.464), but the ‘Real’ group rated murder confessions as more frequent (M = 4.20 on a 6-point scale) than the ‘Fiction’ group (M = 3.55; *t*_77.05_ = 3.43, *p* < 0.001). On balance, we conclude that none of the results relating to rarity could explain the heightened attention and enjoyment experienced by the participants in the ‘Real’ condition.

## Discussion

In a randomised controlled trial, we found little support for hypothesis H1: there was no difference between the conditions in the extent to which participants were transported by the filmed interview. If anything, those who knew it was a real-life interview found it significantly more enjoyable and were more attentive. This might reflect the fact that the film lacked the speed and visual richness we would normally expect from a filmed portrayal. Nonetheless, the fact that there were only limited differences between the two conditions confirms that a fictional story does engage us in broadly the same way as a real-life story (at least when these concern people we do not know). Testing hypothesis H2 confirmed that those who believed the video was fictional were more willing to identify and sympathise with an immoral character, even though they did not necessarily morally approve of his actions, suggesting that a fictive pass did allow some degree of moral licencing (a fictive pass) with respect to a third party. Hypothesis H3 tested whether fiction allows people to take a significantly more balanced view of someone’s actions when they break the moral code. The results suggest that this is not the case: those in the ‘Fiction’ condition were not more willing to take a wider view of why Williams behaved as he did. It may be that this reflected the gravity of Williams’s transgression; if so, it at least confirms the earlier finding by Sabo and Giner-Sorolla (2017) that a fictive pass is granted only to those whose transgressions are relatively mild.

These findings support to the fictive pass hypothesis. This does not necessarily imply that the capacity for storytelling, or the reason why we engage in it so much, evolved to enable the fictive pass (as a way of managing morally complex situations or dilemmas). A more likely explanation is that the fictive pass exploits our enjoyment of storytelling to gain an alternative additional benefit in terms of allowing us to understand complex social behaviour (a form of explanation usually known as a ‘window of evolutionary opportunity’: Dunbar, 2019). That said, our concern here is not to determine *why* we engage in storytelling so much as to understand how this is possible and what consequences it might have. Our findings suggest that fiction does not necessarily allow us to distance ourselves sufficiently from the emotional immediacy of the perpetrator’s behaviour to consider the wider context – the principle on which most legal systems are, of course, based. Whilst we can become engaged in, or transported by, a fictional story, we do not necessarily become so emotionally detached from as to avoid the trap of taking an emotionally extreme view or, in other contexts, of engaging in moral partiality (favouring those who are emotionally close to us). Neither moral partiality nor moral licencing are part of the function of fiction.

We found no evidence to suggest that those who believed murders to be more (or less) frequent also enjoyed the video more. That might be because the video shows real-life come-uppance—a form of the *Schadenfreude Effect* (Singer et al., 2006; Cikara and Fiske, 2012). Those in the ‘Real’ (vs. ‘Fiction’) condition believed that interrogation videos were more widely available to the public, and that murder confessions happened more often than is actually the case. It is possible that information from the video might have updated participants’ beliefs about real-world frequency more in the ‘Real’ condition than in the ‘Fiction’ condition. Taken together, however, these results suggest that it is perhaps not the perceived rarity of the information source as such, but rather the utility of the information, that may be driving the effect—although without an experimental manipulation to test between these two hypotheses directly we cannot be sure.

The modest sample sizes and multiple comparisons might invite caution. Our design was powered only to detect relatively large effects (see Methods), and our multiple outcome measures might increase the risk of false positives. Nevertheless, our results do provide some evidence that there may indeed be systematic differences in how viewers respond to films they believe are real vs. fictional. That said, our results are in agreement with the broader findings of Sperduti et al. (2016), who suggested that knowing a film clip is fictional dampens emotional responses, especially for positive emotions. We here extend their findings by exploring some of the psychological processes, notably emotional identification with specific characters and attribution, that might underpin these effects. A second issue is that we only used one film clip, and our conclusions would inevitably have been more robust had we used two. Suitable film clips were extremely hard to find since they needed to be plausibly both real and fictional at the same time. In addition, we required film clips of significant length in order to allow the viewer to become immersed in the action. Short clips lasting a few minutes might have been easier to locate, but would surely have been much less immersive and hence less likely to produce any kind of effect.

Notwithstanding these limitations, the main purpose of our experiment was to determine whether fiction allows audience members to become more involved with the protagonists in a story without feeling obliged to adopt a strictly moral stance in the way one might in real life. Can we stand back, as it were, and examine the dilemma of the story from all sides because we know it did not really happen? In effect, does fiction give us licence to consider more sympathetically the behaviour and motivations of those who contravene against social norms, to see the world through the eyes of someone who, perhaps because they are trapped by circumstances, behaves in a way we might not normally approve of? Being able to interpret how someone might have come to be trapped in circumstances beyond their control also allows us to evaluate the complex dynamics of the social world in which we ourselves live, and hence, perhaps, prepare ourselves for a more appropriate response should we ever find ourselves in similar circumstances. This may also be crucial in allowing us to learn how and when to make allowances for others in ways that will impact less on the delicately balanced stability and cohesion of both our own personal relationships or that of the wider community—in other words, to apply the rules of society in a more nuanced (and perhaps more humane) way that might have a beneficial effect on the social stability of the community. Though there were some significant hints that this might be so, the evidence was far from clear-cut. In one respect, the results suggest that we do find stories entertaining, but it may not matter whether these are true stories (‘gossip’: Dunbar, 2004) or fiction—we gain a psychological lift from them either way.

In the present case, our results suggest that the ‘fictional’ version was less engaging (i.e., participants reported that their minds wandered more) than the ‘real’ version of the film. This may simply be because real life events lack pace (we may often pause to mull over our reply, especially if it risks incriminating us) and usually take place over a considerable span of time (days rather than hours). The listener (or viewer) quickly gets bored and distracted when viewing things in real time. This may make a real time recounting of events tedious (as when someone recounts every cut and thrust of something that happened to them) instead, we need to cut to the chase and get to the denouement as quickly as possible, whilst at the same time using devices such as ambiguity to heighten the tension and generate interest. This may require the storyteller to foreshorten the action in order to maintain the listener’s (or viewer’s) interest and engagement (as, for example, in G.R.R. Martin’s manipulation of the temporal sequence of events in *A Song of Ice and Fire* [aka *Game of Thrones*]: Gessey-Jones et al., 2020). Inevitably, this was lacking in our excerpt from the original videos, and it may explain why so many of the original ‘Fiction’ participants came to the conclusion that the film was actually a real police interview.

These findings raise an issue of some psychological importance. Being able to maintain the coherence of a storyline and its characterisations whilst physically fully embedded in the conventional real world means being able to run two ‘realities’ simultaneously side by side without them leaking into each other. How the brain manages to toggle between real world social interactions and virtual or imagined social world interactions is an important question (Schilbach et al., 2010). It is not at all obvious how the brain does it. We should not underestimate how cognitively difficult this actually is and it is worth briefly considering the demands involved so as to appreciate the scale of work that our brains do.

In an fMRI imaging study, Viard et al. (2011) showed that, compared to thinking about actual past events, thinking about an imaginary future required more activity in the inferior frontal, lateral temporal gyri and parietal regions—most likely, they suggest, because it involves an imaginative mental construction as opposed to simple recall. It is significant that it is precisely these regions that are involved in mentalising (Carrington and Bailey, 2009; van Overwalle, 2009). This is the same brain network that allows us to manage our social relationships, something we do by creating mental models of other people’s minds abstracted from indirect observable behavioural cues (Powell et al., 2012).

It has been suggested that the right temporoparietal junction acts as a key switching relay between two alternative viewpoints: those that are firmly anchored in the current sensory environment and those that are stimulus-independent and rely exclusively on internally generated information (Bzdok et al., 2013; Kernbach et al., 2018). Since the default mode network seems to underpin the capacity to mentalise, this neural pathway may help mediate shifts of focus from the person in front of us to someone who is not physically present (a capacity that is also essential for thinking about the mindstates of real individuals who are physically present). It seems that it is this cognitively and neurally expensive network that is called on when we become immersed in fiction.

## Data availability statement

The datasets presented in this study can be found in online repositories. The names of the repository/repositories and accession number(s) can be found in the article/Supplementary material.

## Ethics statement

The studies involving human participants were reviewed and approved by University of Oxford Combined University Research Ethics Committee. The patients/participants provided their written informed consent to participate in this study.

## Author contributions

JT devised the study, ran the experiments, analysed the data, drafted the manuscript, and approved the final version of the manuscript. BT devised the study, ran the experiments, analysed the data, and approved the final version of the manuscript. EE and SD devised the study, ran the experiments, and approved the final version of the manuscript. FB devised the study and approved the final version of the manuscript. LM devised the study, revised the manuscript, and approved the final version of the manuscript. RD devised the study, analysed the data, drafted and revised the manuscript, and approved the final version of the manuscript. All authors contributed to the article and approved the submitted version.

## Funding

This research was funded by the Calleva Research Centre, Magdalen College, Oxford.

## Conflict of interest

The authors declare that the research was conducted in the absence of any commercial or financial relationships that could be construed as a potential conflict of interest.

## Publisher’s note

All claims expressed in this article are solely those of the authors and do not necessarily represent those of their affiliated organizations, or those of the publisher, the editors and the reviewers. Any product that may be evaluated in this article, or claim that may be made by its manufacturer, is not guaranteed or endorsed by the publisher.
