# Supplementary material for: Does believing something to be fiction allow a form of moral licencing or a ‘fictive pass’ in understanding others’ actions?
Source: Front Psychol. 2023 May 15;14:1159866. doi: 10.3389/fpsyg.2023.1159866 (PMC10225679; doi:10.3389/fpsyg.2023.1159866)
Supplement: Supplementary file 2 [file Data_Sheet_2.PDF]

Distinguishing Between Reality and Fiction

Jacqueline Thompson, Ben Teasdale, Evert van Emde Boas, Felix Budelman, Sophie Duncan, Laurie Maguire & R.I.M. Dunbar

SUPPLEMENTARY INFORMATION

Summary of the action sequence in the video clip

The interview is skilfully and nonconfrontationally managed by the detective, Smyth. It begins with friendly, neutral chitchat of the kind that might occur between a GP and patient as the two men sit and settle themselves. The detective then offers evidence from tyre tracks and footprints: 'Your vehicle drove up the side of Jessica Lowell's house…. your boots walked to the back of Jessica Lowell's house', but the distancing tactic (it is the vehicle and the boots that have agency) means that the suspect is not directly accused. Smyth frequently invokes the "experts" making it clear that it is they, not he, who make the link between Russell and the crime scene, and that it is the impartial evidence, not a partisan individual, that will decide Russell's guilt or innocence.

The interviewer avoids displays of power. In fact, he presents himself in a supportive role throughout – he wants to give Russell "the benefit of the doubt" and he offers him the "opportunity to take some control here" by telling the truth. His questions are rhetorical or open-ended – invitations rather than accusations. When he asks "What are we going to do?" the first-person plural conveys that he and the accused are a cooperative unit. Despite the suspect's hesitations and long pauses, and despite the tension, the atmosphere is collegial: the suspect tells the detective, "Call me Russ, please" (Smyth accepts this invitation), and Smyth compassionately acknowledges that Russell's mind must be "racing" because "I've seen a lot of people in your position".

That said, the pressure mounts with the detective's drip-release of facts: that there is no limit to the money available for investigation, that computer data can never be permanently erased, that a search team is currently tearing the suspect's wife's house apart, that the search is closing in and the end "might even happen tonight". One of the turning points comes with Smyth's astute question, presented as an offer of help: "Can you tell me what the issue is you are struggling with?" Russell offers two surprisingly non-self-centred answers: he's concerned about the emotional impact on his wife (Smyth: "So am I"; once again, he and Russell are on the same side) and he is concerned about the reputational impact on the Canadian Armed Forces. One can see why this video makes such gripping material: Russell may be a murderer but he is not, as the detective assures him , "a cold-blooded psychopath".

The storyline culminates in a 23-second pause after the detective's final question – "So where is she?" [the dead victim's body], a pause that is finally broken by Russell's dramatic "You got a map?", a theatrical *volte face* worthy of any TV police drama.

Experimental Primes

Reality prime text:
You will now watch a video clip from police CCTV.
It shows a real-life police interrogation of a Canadian military colonel suspected of murder in 2009.
The case received a large amount of local media coverage at the time, and the interrogation recordings were made publicly available on YouTube after the trial by the Canadian police, as part of a transparency initiative. This clip is an edited version of the longer interrogation, which lasted several hours.

Fiction prime text:
You will now watch a clip from a crime drama web series.
The scene depicts a police interrogation in which a Canadian military colonel is suspected of murder.
The clip was taken from "The Interrogation," an original web series which creates a realistic feel by using techniques such as fixed-camera (CCTV-style) shooting, as well as asking the actors to improvise their scenes from a minimal script.
Although the clip is fictional, it includes topics which may be upsetting to some viewers, so please ask the researcher if you are concerned about the content.

Cast List:
James Paul Cooper ... (playing the character of Russell Williams)
Oliver Tomlinson ... (playing the character of Detective Smyth)
Written and directed by Mark Mitchell. With permission from Fair Weather Productions, 2017.

IDENTIFICATION [Cohen 2001 and Tal-Or & Cohen 2010)**:**

On Likert scale (1=strongly disagree; 7=strongly agree) for both Russell and Detective:

- I think I understand […] well
- I understood the events in the scene the way […] understood them
- While viewing, I felt like […] felt
- While watching, I could really ‘‘get inside’’ […]'s head
- I tend to understand why […]l did what he did
- I approve of […]'s behaviour before the interview
- I approve of […]'s behaviour during the interview

For the purposes of analysis, we excluded the last two questions because they partially overlap with the questions on moral approval (see below) that we wished to ask more explicitly.

Enjoyment (adapted from Dunbar et al. 2016)**:**

On a Likert scale (1: strongly disagree; 7: strongly agree):

- I enjoyed the video very much
- Given the opportunity, I would watch this video again
- I would recommend watching this video to my friends
- I am interested in watching the full video this clip was taken from

Moral approval:

Two simple factual questions asking whether participants approved of Russell, on a 7-point Likert scale (1: strongly disagree; 7: strongly agree):

- I approve of Russell's behaviour [that he was a murderer]
- I approve of Russell's behaviour during the interview

Moral compass:

Again, a set of simple factual questions asking whether participants thought it was morally appropriate to engage with real-life and fictional murderers, each on a 7-point Likert scale (1: not at all; 7: very):

- How morally acceptable is it to identify with a real-life person who is a murderer?
- How morally acceptable is it to identify with a fictional character who is a murderer?
- How morally acceptable is it to feel sorry for a real-life person who is a murderer?
- How morally acceptable is it to feel sorry for a fictional character who is a murderer?
- How morally acceptable is it to enjoy watching video about a real-life person who is a murderer?
- How morally acceptable is it to enjoy watching video about a fictional character who is a murderer?

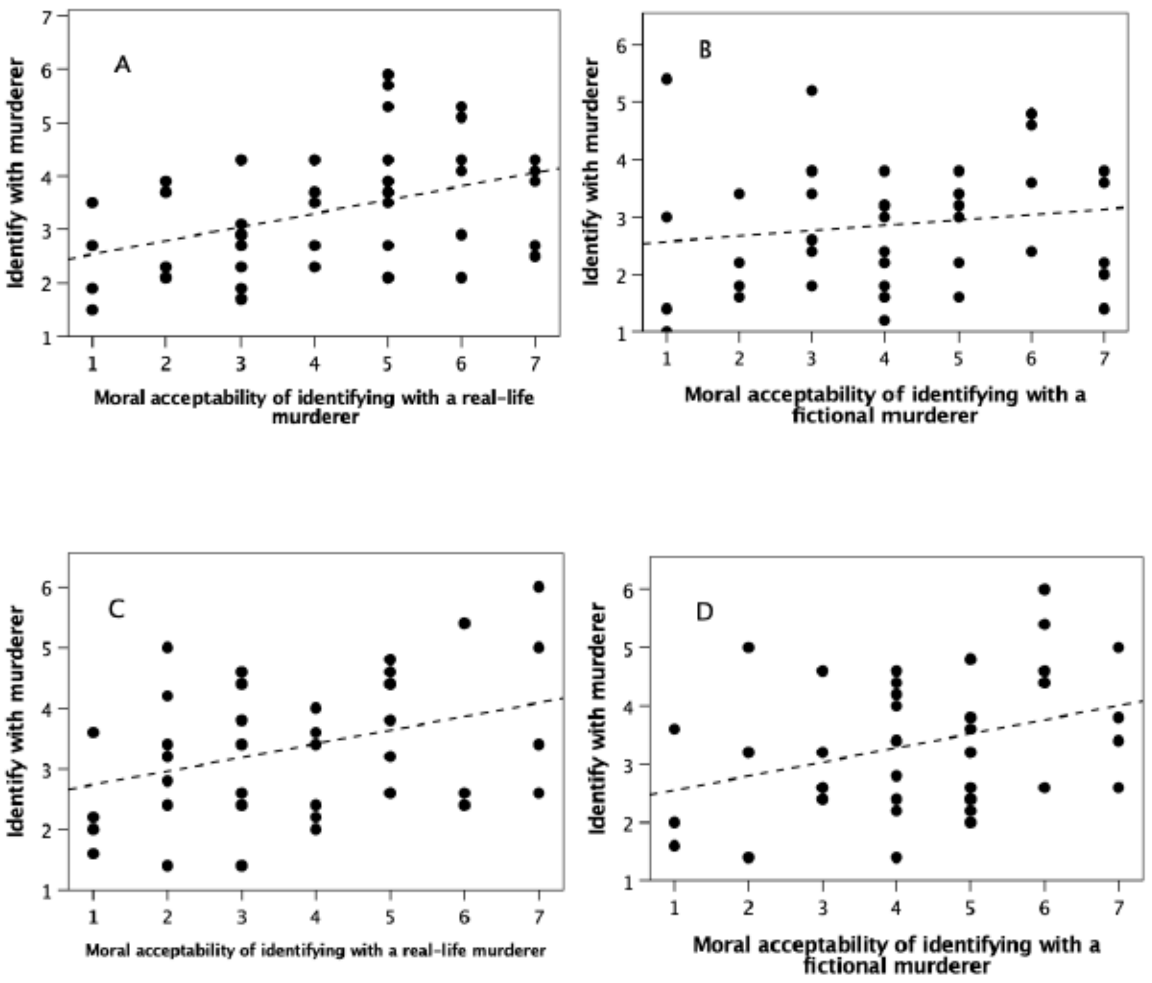

Figure S1
Correlation between extent of identifying with the murderer (Williams) and the rated moral acceptability of identifying with real (a,c) or fictional (b,d) murderers under the ‘Real’ (a,b) and ‘Fiction’ (c,d) conditions.

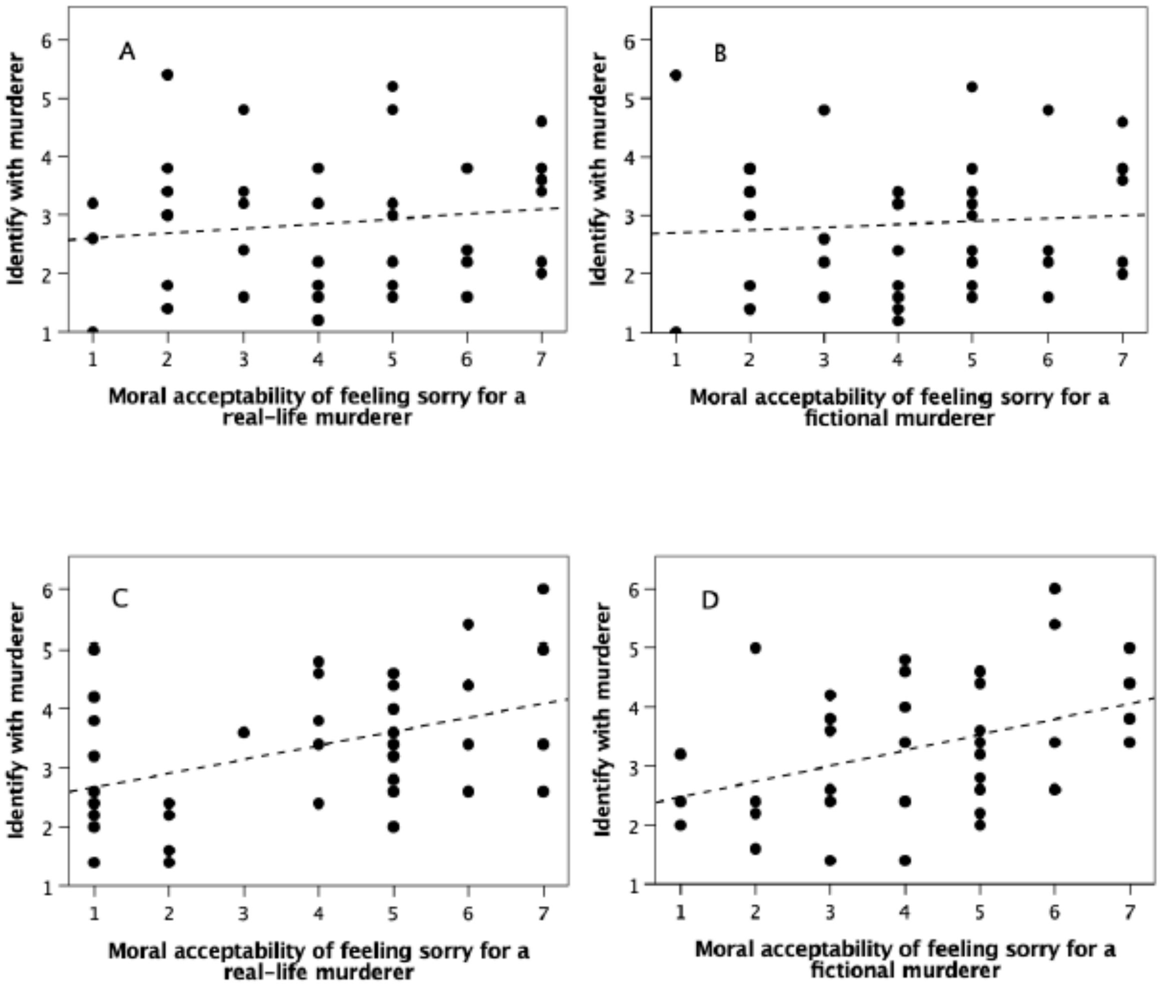

Figure S2
Correlation between extent of identifying with the murderer (Williams) and the moral acceptability of feeling sorry for a real (a,c) or fictional (b,d) murderer under the 'Real' (a,b) and 'Fiction' (c,d) conditions.
